# Considerations for Expanding Antimicrobial Resistance Surveillance Networks

**DOI:** 10.1093/cid/ciad548

**Published:** 2023-12-20

**Authors:** Patrick Gallagher, Alina Shaw, Amit Bhandari, Nimesh Poudyal, Marianne Holm, William R MacWright

**Affiliations:** Public Health Surveillance Group, LLC, Princeton, New Jersey, USA; Public Health Surveillance Group, LLC, Princeton, New Jersey, USA; Health, Nutrition, and Development Independent Consultant, Nepal; International Vaccine Institute, Seoul, Republic of Korea; International Vaccine Institute, Seoul, Republic of Korea; Public Health Surveillance Group, LLC, Princeton, New Jersey, USA

**Keywords:** AMR, surveillance network, Expanding AMR surveillance network, CAPTURA, Asia

## Abstract

Capturing Data on Antimicrobial Resistance Patterns and Trends in Use in Regions of Asia (CAPTURA) gained insight into the range of national antimicrobial resistance (AMR) stakeholders' long-term visions for AMR surveillance networks. As national AMR networks mature, stakeholders often contemplate adding laboratories to the network to achieve greater representativeness, boost data quantity, or meet other goals. Therefore, stakeholders should carefully select laboratories for expansion based on their goals and several practical criteria. Based on CAPTURA experience, the key criteria a national network may consider when expanding its AMR surveillance network include location, laboratory ownership, access to linked clinical and prescription databases, logistical ease, a laboratory's collaborative spirit, laboratory practices and equipment, laboratory staffing and quality assessments, laboratory methods and specimen types, data cleanliness and completeness, and the quantity of AMR data.

## Teaser

Expanding AMR surveillance networks is beneficial. However, stakeholders must think carefully about the criteria for expansion. Based on Capturing Data on Antimicrobial Resistance Patterns and Trends in Use in Regions of Asia's experience, we suggest those criteria.

## EXPANDING ANTIMICROBIAL RESISTANCE SURVEILLANCE NETWORKS IS A WORTHY GOAL

Combatting the rising threat of antimicrobial resistance (AMR) will require increasing the quantity and quality of AMR data to inform decision-making. One method to boost the geographical representativeness and quantity of data available at the national and subnational levels is to expand the number of microbiology laboratories that participate in AMR surveillance networks. In some countries, there are hundreds of microbiology laboratories (both public and private) conducting antimicrobial sensitivity testing (AST) to identify AMR trends, so prioritization and selection of microbiology laboratories to add to national surveillance networks becomes an important decision. Capturing Data on Antimicrobial Resistance Patterns and Trends in Use in Regions of Asia’s (CAPTURA’s) experience in Asia has given the consortium unique insight into criteria that national and subnational stakeholders should consider when expanding an existing AMR surveillance network.

Current AMR networks in Asian countries are often operated by one of several entities: an AMR coordination committee led by the ministry of health, a national reference laboratory within the ministry of health, or a group of research laboratories (public or private) that were established before a national AMR surveillance network was formalized or in a country where a ministry of health does not engage in AMR surveillance. The networks vary in size from a handful of laboratories to several dozen, and their laboratories are often located near urban centers. Most networks strive to aggregate data on a monthly, quarterly, or (most frequently) annual basis and share analysis with national AMR stakeholders and the World Health Organization Global Antimicrobial Resistance and Use Surveillance System (GLASS) [1]. Nearly all networks desire, now or in the future, to expand network membership to enhance data quality and quantity, allowing stakeholders to make more informed decisions.

The benefits of expanding the number of laboratories involved in national AMR surveillance include enhancing outbreak detection, strengthening the capacity of local laboratories, increasing the quantity of high-quality data to inform policy and practices, and improving geographical and socioeconomic representativeness. The involvement of laboratories in surveillance also bolsters the network’s ability to respond to identified threats.

For the above to hold, especially in settings with a large number of microbiology laboratories, the selection criteria for which laboratories to include in surveillance networks become critical. Based on CAPTURA’s experience, key criteria that should be considered in expanding AMR surveillance networks are presented below, but each country must tailor criteria to its goals and desired outputs.

## CRITERIA FOR EXPANSION OF AMR SURVEILLANCE NETWORKS (FIGURE 1)

### Location

A network may consider filling geographic gaps. Laboratories near busy border crossings, in underserved areas with evidence of unusual AMR activity, or near specific animal or environmental exposures may be a priority. A network may also consider including laboratories that serve patient populations across the socioeconomic spectrum.

### Laboratory Ownership

A network may wish to involve a more significant number of privately owned or nongovernmental organization–operated laboratories to enhance quality, broaden the network stakeholder base, or gain exposure to best practices. Adding laboratories associated with academic institutions may also have advantages. Consider offering additional training and robust data privacy agreements to increase the desirability of their participation.

### Access to Linked Clinical and Prescription Databases

Including laboratories that can connect their AMR databases with clinical or prescription databases (especially in-patient, as records are often of higher quality) within the same facility would significantly enhance the richness of the network’s data. Linking AMR data with clinical outcomes allows for the calculation of burden estimates. In addition, connecting AMR data with prescription data enables analysis of prescription appropriateness, antimicrobial stewardship, and treatment guidelines.

### Logistical Ease

Working with large quantities of incoming data requires minimizing bottlenecks. For example, much microbiology data exist as paper records in logbooks or patient files. Investing the time and effort to digitize those records is costly and time-consuming. Therefore, a network may prioritize facilities that already have digitized AMR data. Laboratories that use laboratory information systems (eg, similar metadata, exports into compatible file types) that are compatible with those used by the network are excellent candidates for expansion. For example, WHONET is a software commonly used for GLASS submission; it has the functionality to adapt to specific network requirements.

### Collaborative Attitude

Many networks rely on the willingness of partner laboratories to share data since legal mandates are often weak or nonexistent. The network should consider the collaborative nature of laboratory and facility leadership and be willing to offer incentives for participation. Common nonmonetary incentives include credit in publications, technical training, and feedback on submitted data.

### Laboratory Practices, Equipment, and Quality Assessments

A network may prioritize adding laboratories that demonstrate best practices in AST, invest in new equipment or the maintenance of existing equipment, and demonstrate a commitment to quality control. The results of external quality assurance services and internal quality assessments and accreditations may guide a quality assessment. CAPTURA used a Rapid Laboratory Quality Assessment to grade the relative quality of laboratory practices, but more comprehensive microbiology assessment tools also exist. Stakeholders may also consult in-country societies of microbiologists to gain an understanding of perceived quality.

### Laboratory Staffing

A network may prioritize the existence of senior, credentialed microbiologist staff among laboratory leadership. A network should also recognize the central role of data managers within a laboratory as they organize data into the desired format and transmit it onward. Laboratories with staff who have data management training (eg, data cleaning and restructuring in WHONET or other laboratory management information systems) are better equipped to participate than those who do not.

### Data Cleanliness and Completeness

Suppose a typical dataset from the laboratory is made available for review. In that case, the network may consider if the dataset has an acceptable percentage of missing values, unrealistic values, duplicate entries, and similar characteristics. Laboratories with cleaner, more complete data are more likely to enhance a network.

### Underrepresented Methods, Specimen Types, or Wards

A network may wish to add laboratories with certain specimen types or advanced laboratory diagnostics that are underrepresented in the existing network. Similarly, laboratories that serve hospitals with specific specializations (eg, infectious disease, sepsis) may be desirable, depending on the network’s goals.

### Quantity of Data

Quality issues being equal, a network may prioritize adding laboratories with more significant amounts of data over laboratories with smaller quantities of data.

Other criteria may exist, depending on a network’s context and goals. However, based on CAPTURA’s experience, we recommend that network expansion occur gradually, focusing on data quality and timeliness over quantity, regardless of the criteria that stakeholders choose.

Importantly, we note that these same criteria may apply to expanding AMR surveillance networks in the animal and environmental sectors.

AMR is an ongoing challenge that requires long-term vision from network leadership and other stakeholders. We hope our CAPTURA experience with surveillance networks contributes to shaping that vision.

**Figure 1.**
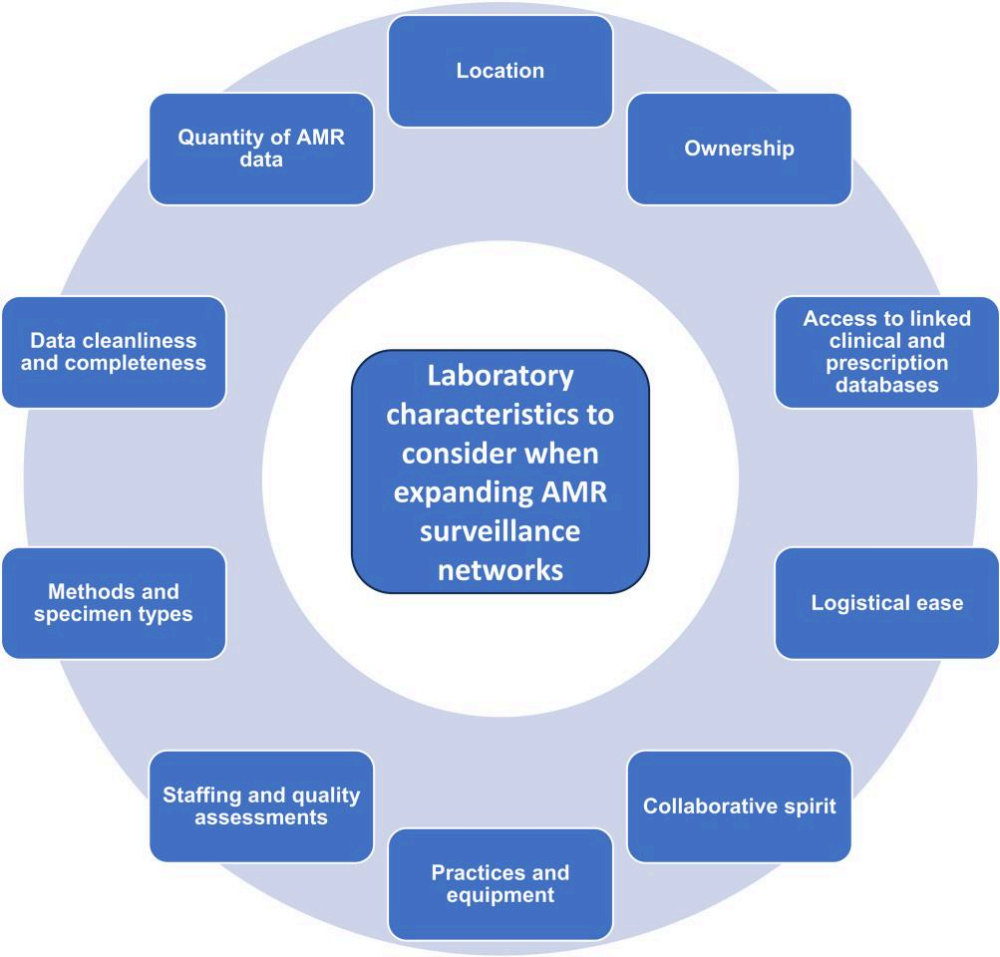
Criteria for expanding AMR surveillance networks. Abbreviations: AMR, antimicrobial resistance.

## Notes

***Disclaimer.*** The views expressed here are those of the authors and not necessarily those of the UK Department of Health and Social Care or its management agent, Mott MacDonald; and, International Vaccine Institute.

***Supplement sponsorship.*** This article appears as part of the supplement “Capturing Data on Antimicrobial Resistance Patterns and Trends in Use in Regions of Asia (CAPTURA),” funded by the Department of Health and Social Care's Fleming Fund using UK aid.

***Financial support.*** This work was supported by the Fleming Fund regional grants (grant numbers FF10-135 and FF11-139). The Fleming Fund is a UK aid investment to support surveillance of antimicrobial resistance in low- and middle-income countries in Africa and Asia. The regional grants program is managed by Mott MacDonald Ltd, the Fleming Fund Grants Management Agent.
